# Supplementary material for: Confirmation bias through selective readout of information encoded in human parietal cortex
Source: Nat Commun. 2025 Jun 25;16:5391. doi: 10.1038/s41467-025-61010-x (PMC12198416; doi:10.1038/s41467-025-61010-x)
Supplement: Supplementary file 1 — Supplementary Information [file 41467_2025_61010_MOESM1_ESM.pdf]

Supplementary Information
Confirmation Bias through Selective Readout of Information Encoded in Human Parietal Cortex

Hame Park, Ayelet Arazi, Bharath Chandra Talluri, Marco Celotto, Stefano Panzeri, Alan A Stocker & Tobias H Donner

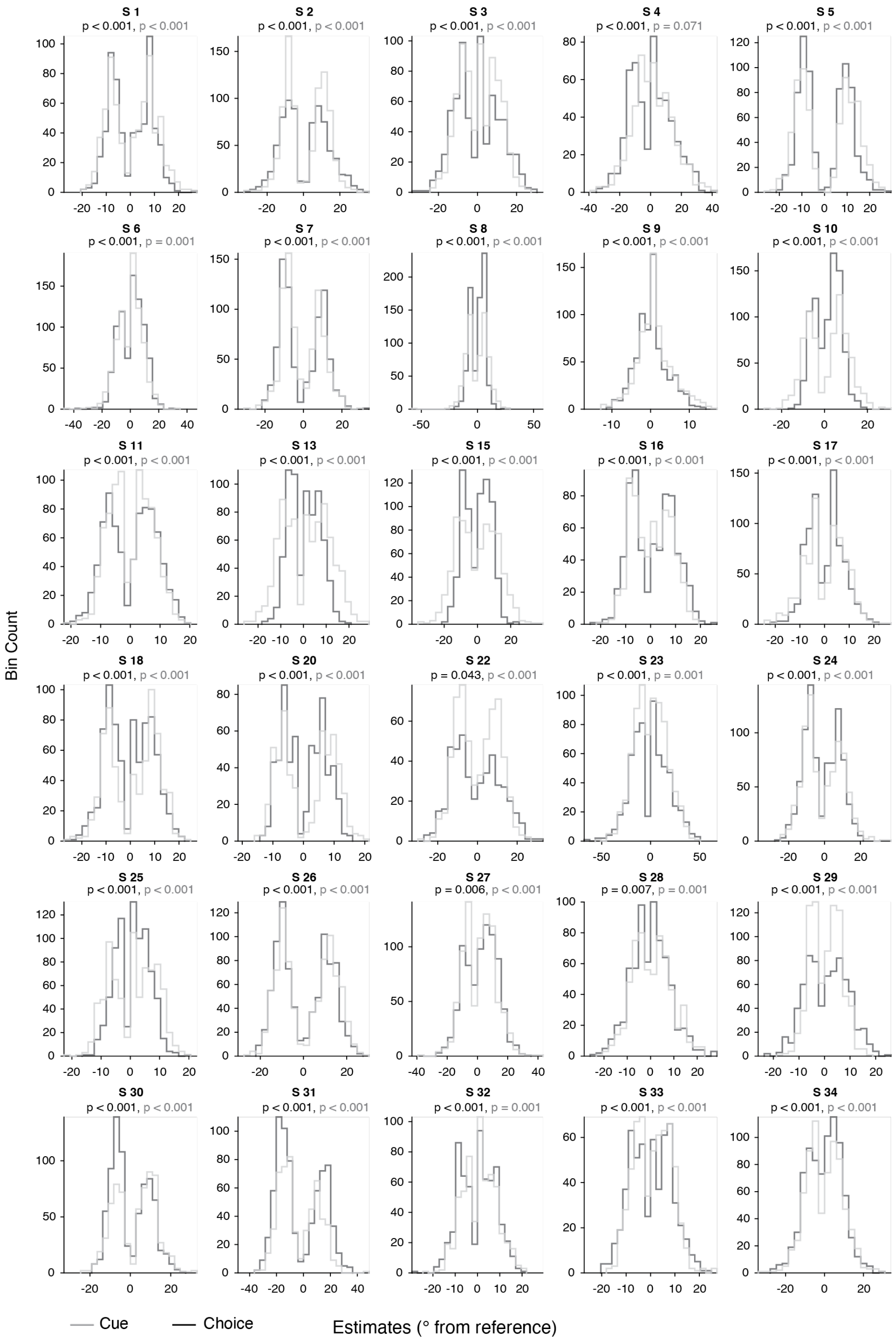

Supplementary Figure 1. Individual distributions of estimation reports.
Individual estimation distributions collapsed across generative means, for Cue (gray) and Choice (black) conditions. P-values in corresponding colors: Hartigan's dip test of unimodality, showing deviation from unimodality in each particpant for Choice, and all but one participant (S 4) for Cue.

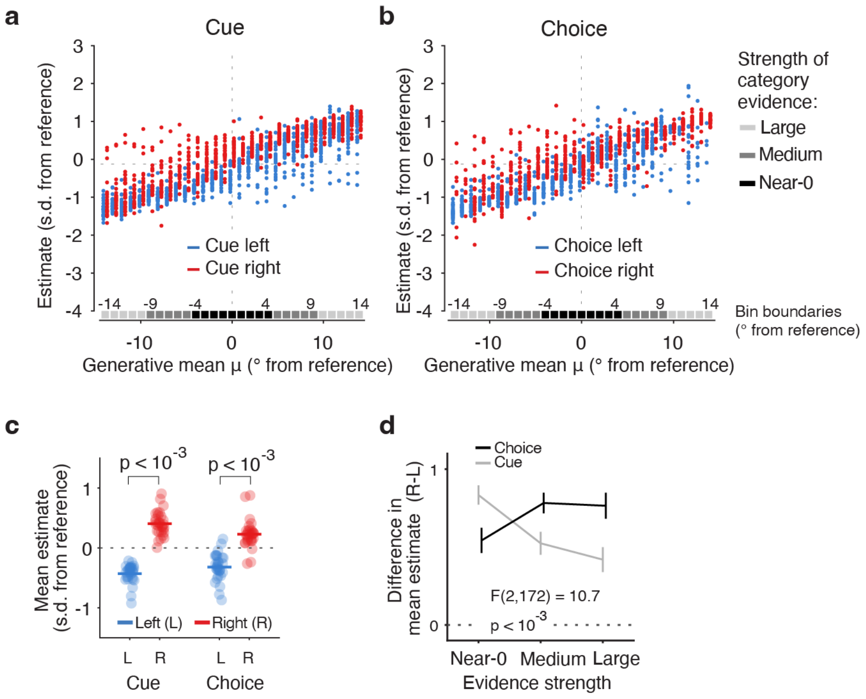

Supplementary Figure 2. Bias in estimation reports.
(a,b) Mean estimation reports as function of generative means, conditioned on the category (left or right) of the cue (a) or the choice (b). Data points, mean estimates for single participants. Cue validity was 75%, based on the generative mean. N = 27 participants. The remaining three participants had a coarser spacing of generative means (steps of 5°) and were therefore not suited for this analysis based on generative mean. **(c,d)** As Figure 1h,i, for sorting of trials based on sample mean as opposed to generative mean. N = 30 participants. (c) Mean estimates for trials with 'right' and 'left' choices or cues. (d) Difference of mean estimates between 'right' and 'left' category trials as in (c) as function of the strength of category evidence, separately for Cue and Choice. Here, the sample mean was computed up to sample 6. This was done to capture the strength of category evidence available when the choice was made, which should most closely relate to the confidence about the choice. Data points, group; error bars, SEM; p-values, two-sided permutation tests (FDR-corrected). Statistics, interaction condition x evidence strength (two-factorial ANOVA, $\eta p^2 = 0.111$, 95% CI: [-0.036, 0.258]).

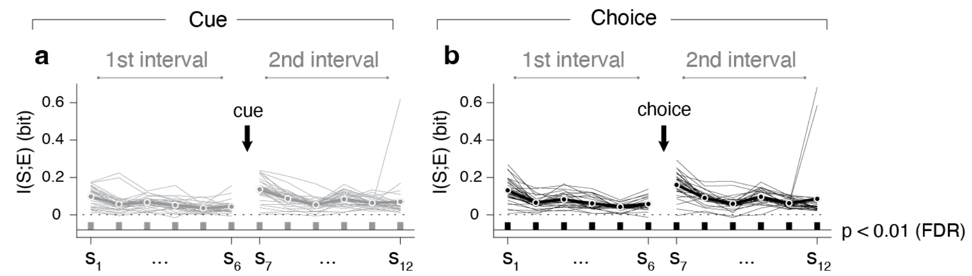

Supplementary Figure 3. Psychophysical kernels using information-theoretic measures. I(S;E), the mutual information between sample (S) and estimation report (E), for **(a)** Cue and **(b)** Choice conditions. I(S;E), quantifies the impact of stochastic trial-to-trial fluctuations of sensory evidence at a given position in the sample sequence on behavioral estimation reports ("psychophysical kernels"[1–3]). Bars below time courses, statistical significance (permutation tests, FDR-corrected, threshold $p < 0.01$). In both conditions, I(S;E) was significantly larger than 0 for all sample positions, consistent with accumulation of evidence across the sequence and with the psychophysical kernels obtained in categorical decisions[1–3]. Note that I(S;E) values computed for all trials are smaller than the averages of I(S;E) values computed separately for consistent and inconsistent samples (Figure 2a,b). This is expected because the information contained in a pooled set of observations with inhomogeneous encoding properties (here: consistent and inconsistent samples pooled) is smaller than the average of the information contained in each set[4]. N=30 participants for all panels.

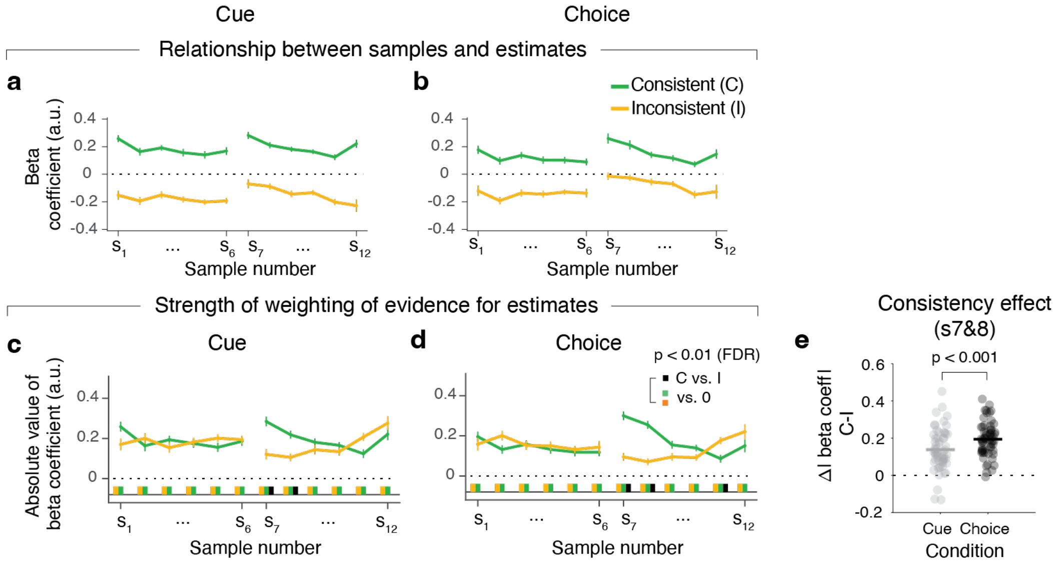

Supplementary Figure 4. Psychophysical kernels evaluated with linear regression.

(a, b) Psychophysical kernels for Cue (a) and Choice (b), computed using linear regression (Methods) for consistent versus inconsistent samples. Regression coefficients have opposite signs for consistent and inconsistent stimulus samples because they also tend to have different signs with respect to the generative means. This is to be expected from the statistics of the experiment (choices and cues, which define consistency, are correlated with generative mean). What is not predicted from the statistics of the experiment is a difference in the magnitude of the regression coefficients (i.e., absolute value, or distance from dashed horizontal line at y=0) for consistent and inconsistent samples. For comparison, see simulation results from Supplementary Figure 5 (left-most column), showing that such differences in magnitude require additional mechanisms that deteriorate the contribution of inconsistent samples to the estimate. Because mutual information I(S;E) from main Figure 2 measures the strength of the association between S and E, regardless of the sign of this association, the absolute value of the regression coefficients is also the linear analogue of I(S;E). **(c, d)** Absolute values of regression coefficients from a, b. **(e)** Comparison of consistency effect between Choice and Cue (from panels a and b), for samples 7 and 8 pooled.

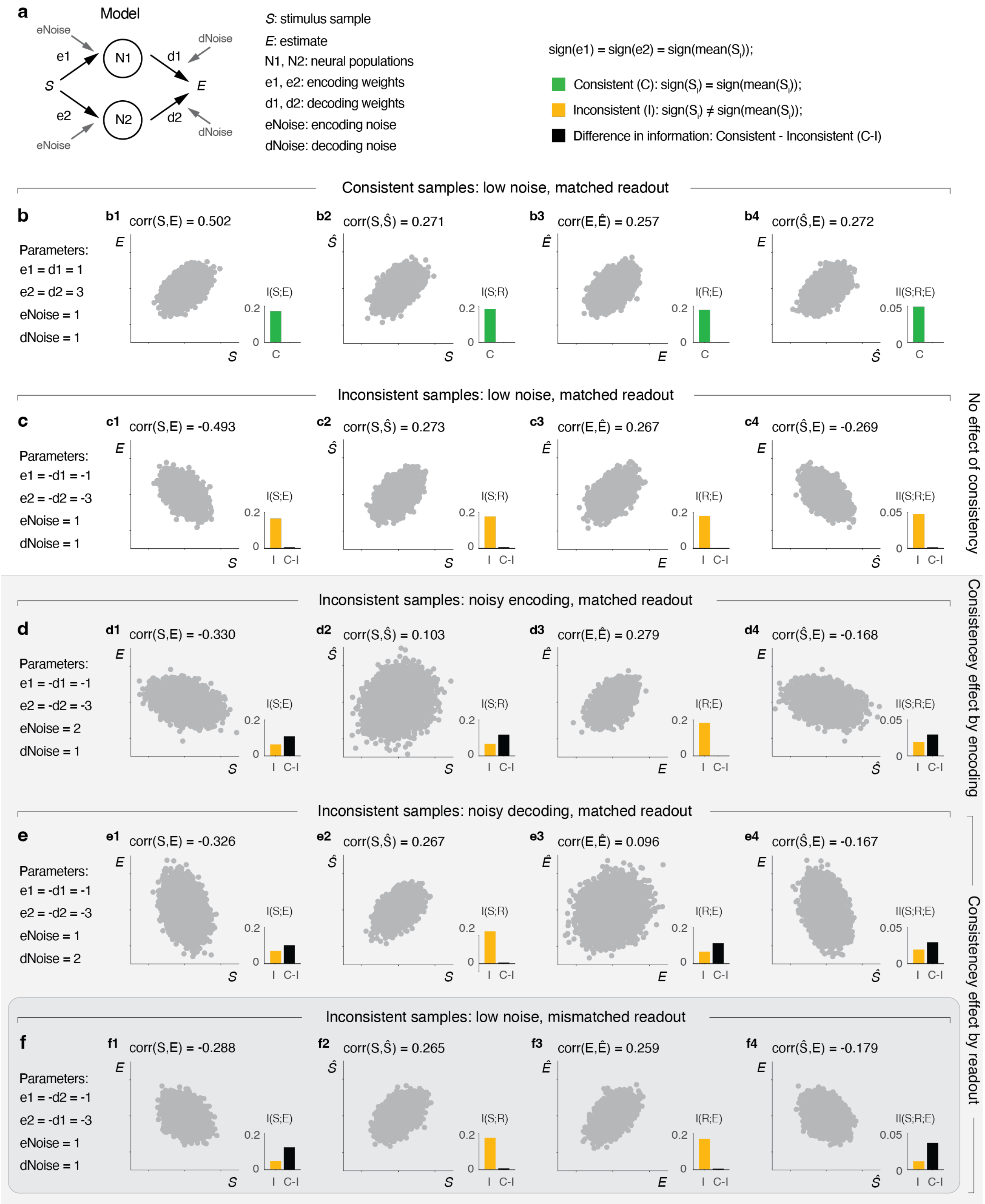

Supplementary Figure 5. Simulation of candidate neural mechanisms underlying selective evidence weighting.

We simulated a simple encoding and decoding model transforming stimulus samples into estimates under various scenarios, to pinpoint alternative hypotheses for the neural computations giving rise

to consistency effects in behavioral evidence weighting as measured by the psychophysical kernels (Methods, section *Information-theoretic and linear analyses of simulated behavior and neural activity*). We used the model to derive predictions for a set of measures (information-theoretic and linear) to quantify neural information encoding and readout, to be assessed in our MEG data. **(a)** Model schematic. Stimulus samples are encoded linearly in the strength of neural activity in two neural populations, and the resulting neural activity pattern is then decoded to produce an estimate, by applying linear weights to the activity of each population. Encoding and decoding are both corrupted by separate sources of noise. The signs of encoding weights are matched to the sign of the *typical* sample (i.e. sign of the mean of many samples), and the consistency of a given stimulus sample is defined by the correspondence of its sign with that typical sample sign. **(b)** Model predictions for consistent samples, without information loss, with good readout. **(c)** Model predictions for inconsistent samples, without any information loss. This case is only shown for reference, since it does not produce any effect of consistency on psychophysical kernels, measured via the linear corr(S,E) or mutual information I(S;E). **(d-f)** Three candidate neural mechanisms for reducing the impact of inconsistent samples on the estimate (gray background). In the first case (d), there is high encoding noise but the readout is still good. In the second (e) and third (f) cases, the readout is suboptimal, in two different ways: high decoding noise, or (without any change in encoding or decoding noise), the encoding and readout weights are mismatched. Importantly, each of these three cases gives rise to the observed effect in the psychophysical kernels (less contribution of inconsistent samples to estimate). The third case (mismatched readout, panel f, darker gray background) produces the pattern of neural measures we observe in our data for the information theoretic measures and linear measures of neural encoding and readout.

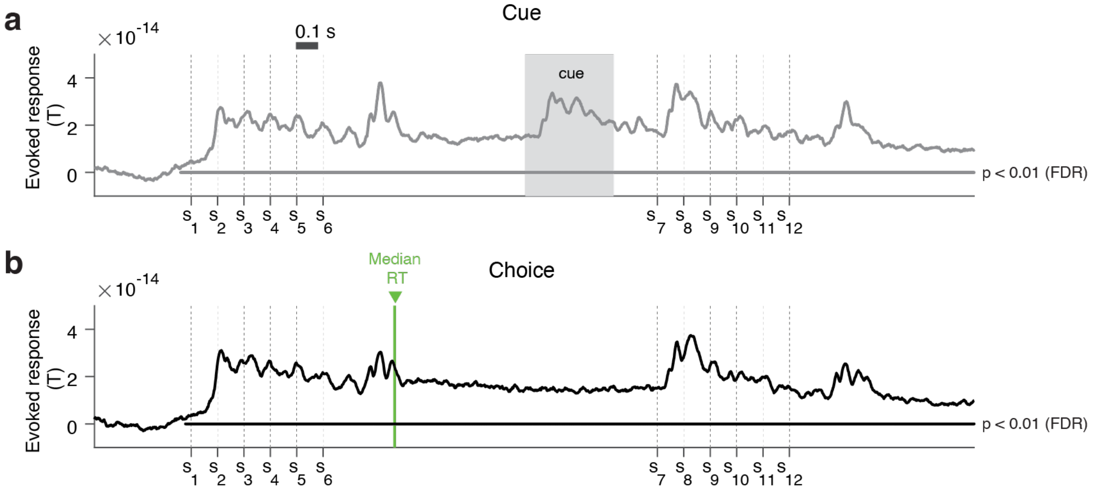

Supplementary Figure 6. Evoked response in dorsal visual cortex.
Time course of trial-averaged response of dorsal visual cortex, for Cue (a) and Choice (b) tasks. Absolute values of responses from each vertex in the area group (Methods) were extracted before averaging to preclude cancellation due to different polarities, and the average activities in the baseline interval (0.5 s -0 s before onset of stimulus sequence) were subtracted from the entire trial. Grey shaded area in (a) is the display period of the categorical cue, and the green vertical line in (b) is the median reaction time (RT) for the categorical choice.

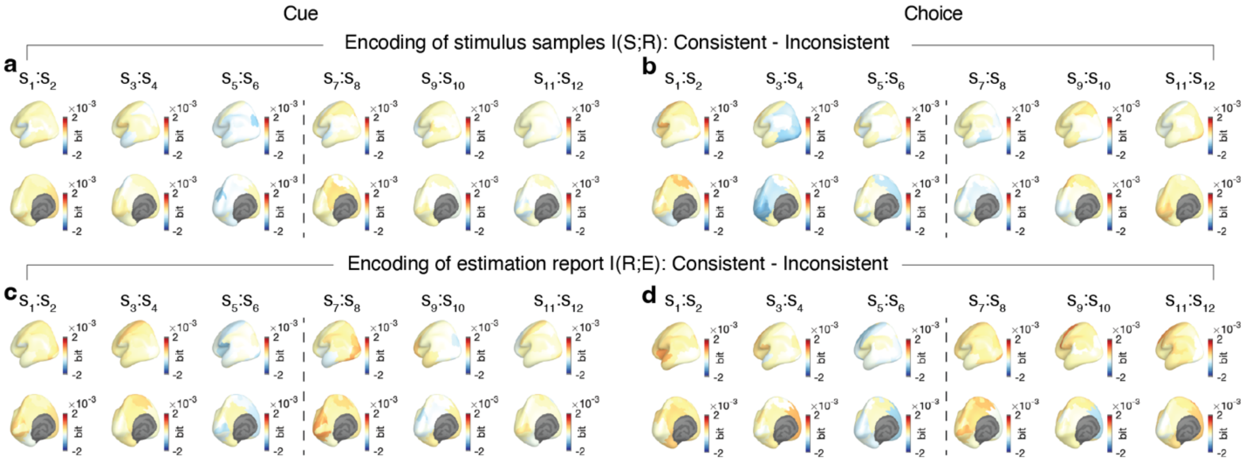

Supplementary Figure 7. No effects of consistency with choice/cue on I(S;R) or I(R;E) across time and space.

Maps of consistency effects (consistent - inconsistent) on information measures across the complete trial (two successive samples pooled, see main text), for Cue (left) and Choice (right) conditions, respectively. **(a,b)** Effects on I(S;R). **(c,d)** Effects on I(R;E). S: sample; R: cortical population response; E: estimation report. The maps are displayed without threshold to appreciate the pattern. None of the effects is statistically significant at any time or in any cortical region, even with liberal thresholds. See also Supplementary Figure 9a-d and Figure 4a-d for the absence of consistency effects on I(S;R) and I(R;E) in the dorsal visual cortex group at sample positions 1, 2 (Supplementary Figure 9a-d) or 7,8 (Figure 4a-d), respectively.

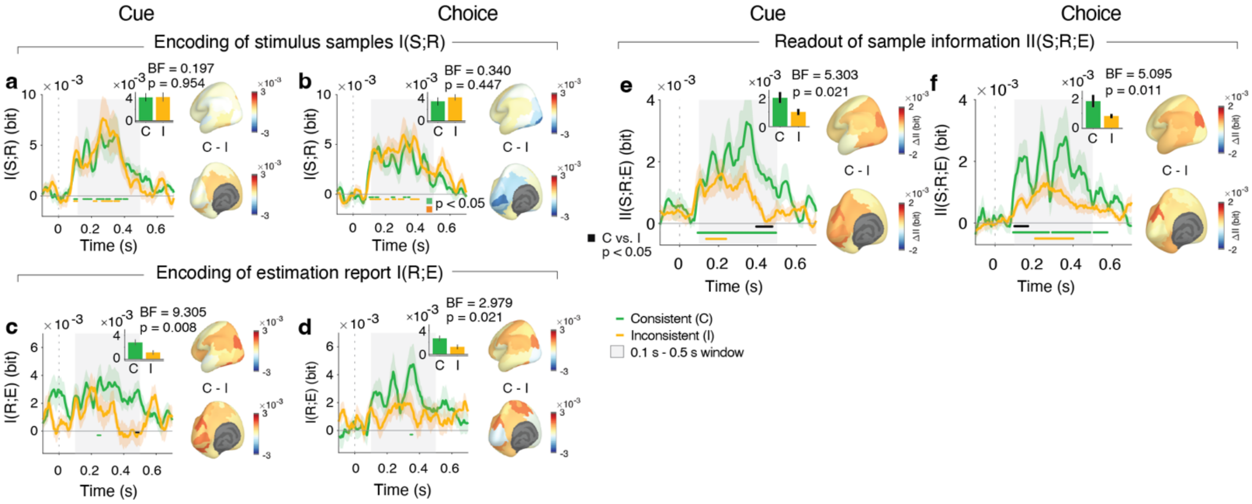

Supplementary Figure 8. Effect of consistency on evidence readout for first principal component.

Effects of consistency on information measures in dorsal visual cortex and across cortex, as in main Figure 4, but now evaluated for the first principal component only. Bar charts in insets: time averaged values across gray-shaded interval. Again, there is a clear effect of consistency on II(S;R;E) quantifying evidence readout, but not such effect on I(S;R) quantifying evidence encoding. N=29 participants.

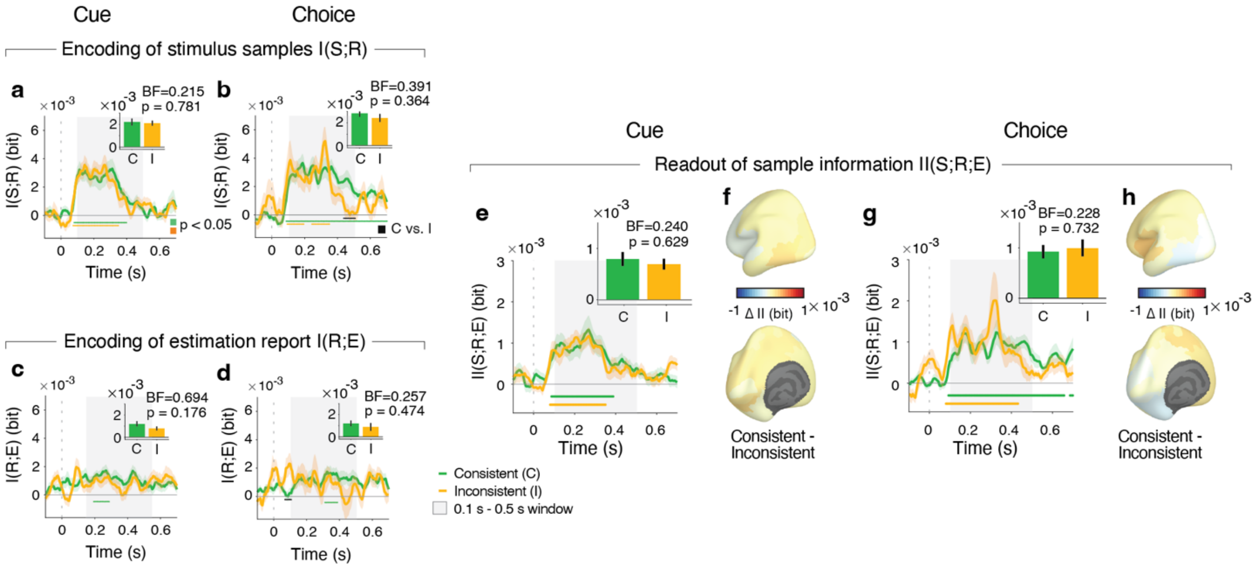

Supplementary Figure 9. No effects of consistency on information measures during first two samples.

As main Figure 4, but for the first two samples of the first trial interval preceding choice or cue. Bar charts in insets: time averaged values across gray-shaded interval. Despite robust stimulus information in both conditions (Cue, Choice) for consistent (C) as well as inconsistent (I) samples, there is no effect of consistency on these measures anywhere, demonstrating the temporal specificity of the effect shown in Figure 4. N ranges between 27 and 29 participants for different panels, contingent on participant inclusion based on the criterion of at least 81 trials (for computation of intersection information) per condition and sample position (Methods).

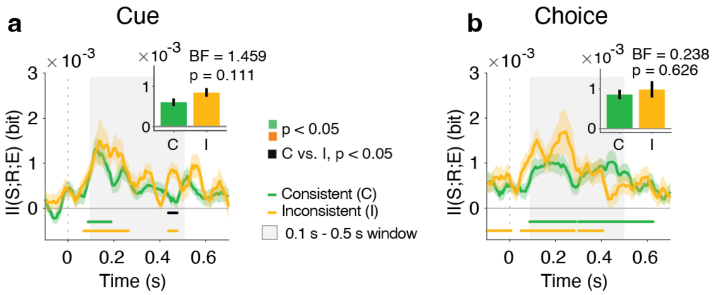

Supplementary Figure 10. No effect of consistency on intersection information just before choice or cue.

As main Figure 4, but for the last two samples of the first trial interval preceding choice or cue. Bar charts in insets: time averaged values across gray-shaded interval. **(a)** Cue condition. **(b)** Choice condition. N ranges between 28 and 29 participants for different panels, contingent on participant inclusion based on the criterion of at least 81 trials (for computation of intersection information) per condition and sample position (Methods).

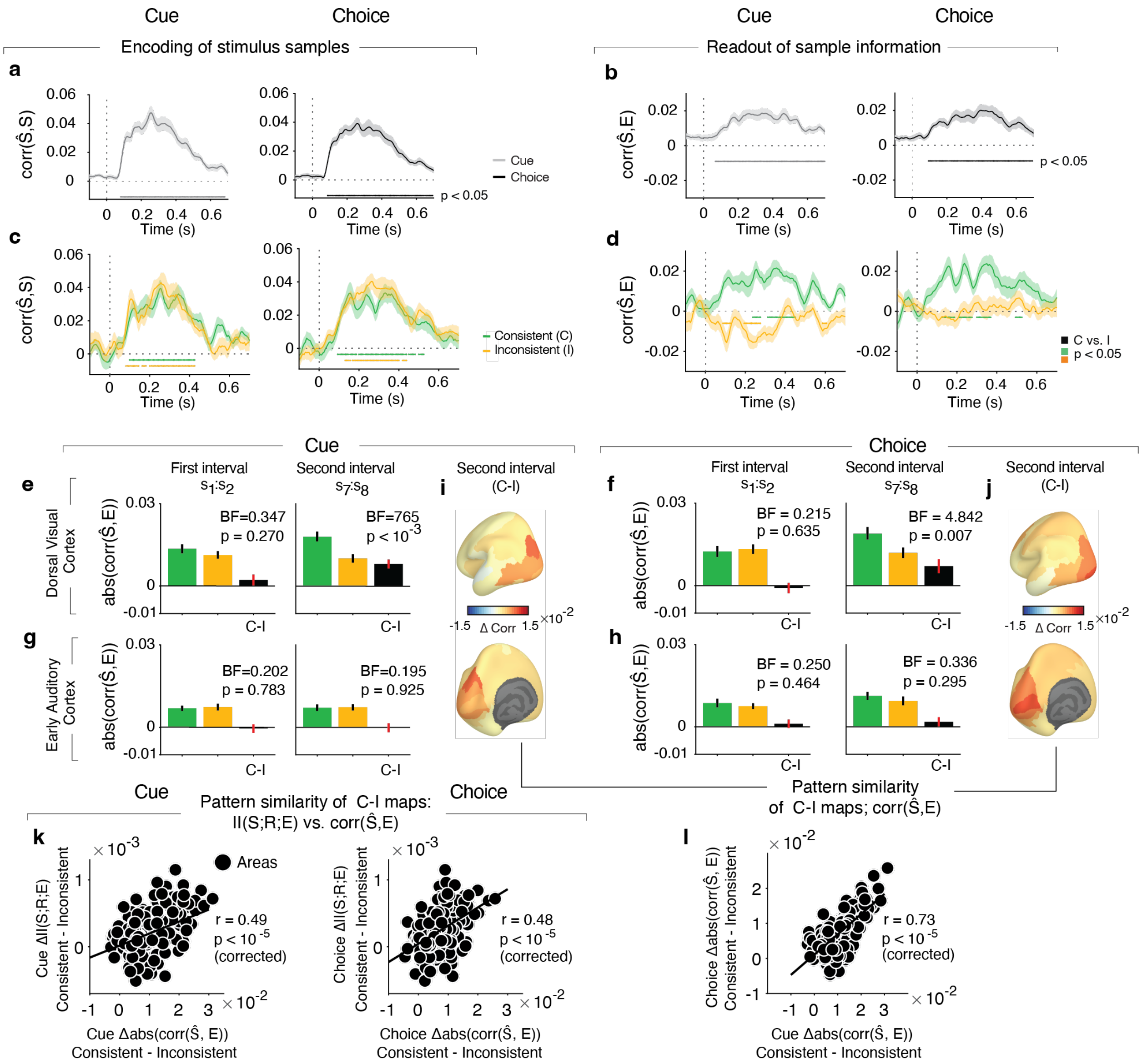

Supplementary Figure 11. Similar effect of consistency on readout as quantified by decoding with linear regressions.

We used cross-validated linear decoding as an alternative approach for quantifying the neural encoding and readout of stimulus samples (Methods). **(a)** Decoding performance for dorsal visual cortex, evaluated as the correlation between stimulus sample predicted from the linear regression of the single-trial neural activity pattern and the actual stimulus samples (samples 7,8 pooled). We find significant correlation, with a time course akin to the one of I(S;R) (Figure 4a,c). **(b)** Linear-regression decoder analogue of intersection information II(S;R;E) for dorsal visual cortex: $\mathrm{corr}(\hat{s}, E)$, the correlation between individual decoder-predicted stimulus samples $\hat{s}$ and the behavioral estimate E (see reference[5] for a more detailed conceptual explanation). **(c,d)** As (a,b), but sorted by consistency of the evidence samples. Color coded dots below time-courses denote statistical significance at $p < 0.05$ from cluster-based permutation tests. **(e)** Absolute values of

correlation between decoder-predicted samples for time interval of maximum stimulus information (0.1 s – 0.5 s post sample onset; same as for main figures) and behavioral estimates, for Cue condition. The magnitude of $\mathrm{corr}(\hat{s}, E)$(i.e., the absolute value of data in (d)) is the linear analogue of intersection information II(S;R;E). Hence, the difference between consistent and inconsistent samples (black bar) is the analogue of the consistency effect on II(S;R;E) in the main paper. Results are shown separately for the first two samples of the first and second stimulus intervals. **(f)** As (e) but for Choice condition. Both conditions show a clear effect for the second interval. **(g,h)** As (e,f) but for early auditory cortex as negative control, showing no consistency effect. P-values for dorsal visual cortex and early auditory cortex from two-sided permutation tests. **(i,j)** Corresponding maps of the consistency effect on the linear analogous of Intersection Information $\mathrm{abs}(\mathrm{corr}(\hat{s}, E))$ in second interval (black bar) from panels (e-h) across cortex, exhibiting clear peaks in early and dorsal visual cortex. N=29 participants; bars or lines, group mean; error bars or shaded areas, SEM. **(k)** Pattern correlations of fine-grained spatial maps of consistency effects on II(S;R;E) versus on $\mathrm{abs}(\mathrm{corr}(\hat{s}, E))$, separately for Cue and Choice conditions. Patterns of cortical consistency effects are highly similar for the information measure and corresponding linear measure of sample readout. **(l)** Pattern correlations between consistency effects on $\mathrm{abs}(\mathrm{corr}(\hat{s}, E))$ in Cue and Choice, revealing high spatial similarity as well. Taken together, these control analyses show similar effects of consistency on neural the readout of stimulus (sample) information from parietal cortex as obtained in the information-theoretic analyses presented in the main paper, including similar effects for Choice and Cue conditions. Data points in all scatter plots are parcels from the Glasser atlas[6] (N=180) and p-values are from spatial autocorrelation-preserving permutation test (see Methods). Abbreviation: BF; Bayes Factor.

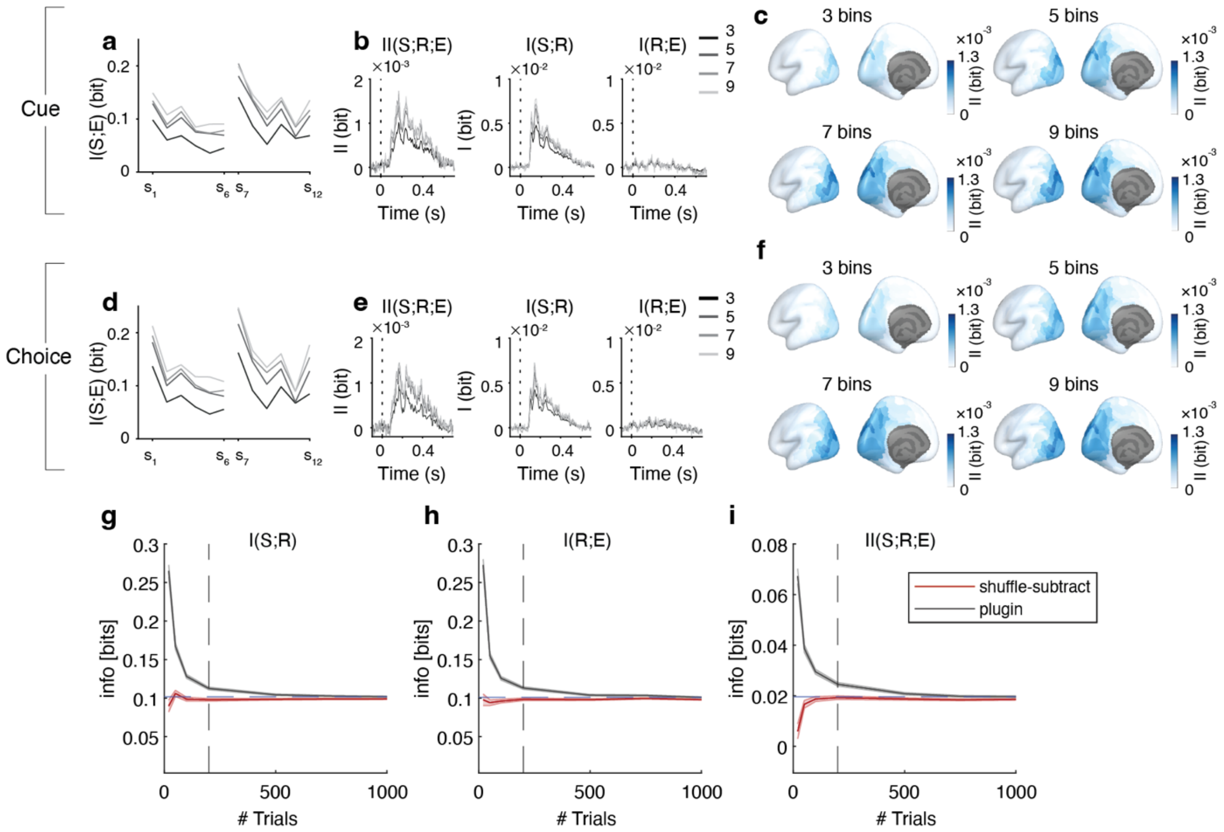

Supplementary Figure 12. Effect of number of bins and trial counts on information theoretic measures.

(a) Information between stimulus (S) and estimation report (E) for different bin numbers. **(b)** Time courses of II(S;R;E), I(S;R), and I(R;E) for different number of bins (dorsal visual cortex). **(c)** Maps of II(S;R;E) on cortical surface for different bin numbers. **(d-f)** As (a-c), but for Choice condition. The qualitative patterns of all information theoretic measures were stable over the range of bin numbers explored, supporting our choice of a bin size of 3, to maximize trial counts for the main analyses of the consistency effects on information measures (see Methods). N=30 participants for all panels. Bars, lines; group averages; error bars, SEM. **(g-i)** Computation of I(S;R), I(R;E) and II(S:R;E) as a function of the number of simulated trials used to compute them when using 3 bins to bin each quantity (exactly as for the real data analysis). Vertical line: average number of trials used for the analyses of real data in this paper (~200). "Plugin": plugging empirical probability histograms into the information equations before using any limited-sampling bias correction. "Shuffle-subtract": information values obtained after correcting for the limited sampling bias with the shuffle-subtraction correction that was used in the real data analysis. Results in each panel are plotted as mean ± SEM over 500 simulations with the number of trials specified in the x axis. The correct value of the information quantities, which can be estimated from very large numbers of trials, was achieved already with smaller number of trials of the order of those used in our paper or even smaller. Thus, accurate information calculations are possible with the number of

trials available in our empirical datasets. The process simulated in each trial consisted of a value of the neural activity drawn from a Gaussian distribution, then a value of sample and estimate obtained by linear convolution of neural activity and adding noise as described in equations (4,5) of the main text.

Supplementary references

1. Murphy, P. R., Wilming, N., Hernandez-Bocanegra, D. C., Prat-Ortega, G. & Donner, T. H. Adaptive circuit dynamics across human cortex during evidence accumulation in changing environments. *Nat Neurosci* **24**, 987–997 (2021).
2. Wilming, N., Murphy, P. R., Meyniel, F. & Donner, T. H. Large-scale dynamics of perceptual decision information across human cortex. *Nat Commun* **11**, 5109 (2020).
3. Waskom, M. L., Okazawa, G. & Kiani, R. Designing and Interpreting Psychophysical Investigations of Cognition. *Neuron* **104**, 100–112 (2019).
4. Kuan, A. T. *et al.* Synaptic wiring motifs in posterior parietal cortex support decision-making. *Nature* **627**, 367–373 (2024).
5. Panzeri, S., Harvey, C. D., Piasini, E., Latham, P. E. & Fellin, T. Cracking the Neural Code for Sensory Perception by Combining Statistics, Intervention, and Behavior. *Neuron* **93**, 491–507 (2017).
6. Glasser, M. F. *et al.* A multi-modal parcellation of human cerebral cortex. *Nature* **536**, 171–178 (2016).
