# Supplementary material for: Confirmation bias through selective readout of information encoded in human parietal cortex
Source: Nat Commun. 2025 Jun 25;16:5391. doi: 10.1038/s41467-025-61010-x (PMC12198416; doi:10.1038/s41467-025-61010-x)
Supplement: Supplementary file 2 — Reporting Summary [file 41467_2025_61010_MOESM2_ESM.pdf]

nature portfolio

Corresponding author(s): Tobias H Donner

Last updated by author(s): May 7, 2025

Reporting Summary

Nature Portfolio wishes to improve the reproducibility of the work that we publish. This form provides structure for consistency and transparency in reporting. For further information on Nature Portfolio policies, see our Editorial Policies and the Editorial Policy Checklist.

Statistics

For all statistical analyses, confirm that the following items are present in the figure legend, table legend, main text, or Methods section.

n/a	Confirmed	
☐	☒	The exact sample size (*n*) for each experimental group/condition, given as a discrete number and unit of measurement
☐	☒	A statement on whether measurements were taken from distinct samples or whether the same sample was measured repeatedly
☐	☒	The statistical test(s) used AND whether they are one- or two-sided<br>*Only common tests should be described solely by name; describe more complex techniques in the Methods section.*
☐	☒	A description of all covariates tested
☐	☒	A description of any assumptions or corrections, such as tests of normality and adjustment for multiple comparisons
☐	☒	A full description of the statistical parameters including central tendency (e.g. means) or other basic estimates (e.g. regression coefficient) AND variation (e.g. standard deviation) or associated estimates of uncertainty (e.g. confidence intervals)
☐	☒	For null hypothesis testing, the test statistic (e.g. *F*, *t*, *r*) with confidence intervals, effect sizes, degrees of freedom and *P* value noted<br>*Give P values as exact values whenever suitable.*
☒	☐	For Bayesian analysis, information on the choice of priors and Markov chain Monte Carlo settings
☒	☐	For hierarchical and complex designs, identification of the appropriate level for tests and full reporting of outcomes
☐	☒	Estimates of effect sizes (e.g. Cohen's *d*, Pearson's *r*), indicating how they were calculated

Our web collection on statistics for biologists contains articles on many of the points above.

Software and code

Policy information about availability of computer code

Data collection	MEG, eye-tracking and pupillometry data were collected using the acquisition software developed by the system manufacturer (MEG: CTF Systems Inc., version 5.4.2; Eye-tracking/pupil: Eyelink 1000, SR Research, version 4.594). Behavioral data were collected using MATLAB version 2014a, using stimulus presentation functions from Psychtoolbox 3.
Data analysis	-MATLAB version 2020b was used for the majority of analyses, including custom code (behavioral analysis/MEG analysis),<br>-The FieldTrip toolbox version 20171001 (MEG preprocessing, including the Infomax algorithm for independent component analysis). E ye-blinks and saccades in the eye-tracking data were detected online by the measurement device (Eyelink 1000) using software from the system manufacturer (SR Research version 4.594).<br>-An In-house pipeline for source reconstruction PyMEG (https://github.com/DonnerLab/pymeg; Python 3.6 in combination with FreeSurfer version dev5-20161028 and the MNE toolbox version 0.16.2) were used for MRI-informed source localization of MEG data.<br><br>-The information theoretic routines implementing all the core analyses used in the paper are available at:<br>Lorenz GM, Engel NM, Celotto M, Koçillari L, Curreli S, Fellin T, et al. (2025) MINT: A toolbox for the analysis of multivariate neural information coding and transmission. PLoS Comput Biol 21(4): e1012934. https://doi.org/10.1371/journal.pcbi.1012934<br>Link to toolbox https://github.com/panzerilab/MINT<br><br>All custom code are publicly available on GitHub (https://github.com/DonnerLab/2025_Park_ConfirmationBias-through-Selective-Readout-of-Information-in-Human-Parietal-Cortex). https://doi.org/10.5281/zenodo.15350393

For manuscripts utilizing custom algorithms or software that are central to the research but not yet described in published literature, software must be made available to editors and reviewers. We strongly encourage code deposition in a community repository (e.g. GitHub). See the Nature Portfolio guidelines for submitting code & software for further information.

Data

Policy information about availability of data

All manuscripts must include a data availability statement. This statement should provide the following information, where applicable:

- Accession codes, unique identifiers, or web links for publicly available datasets
- A description of any restrictions on data availability
- For clinical datasets or third party data, please ensure that the statement adheres to our policy

The behavioral data and preprocessed neural data (as time courses of principal components, information measures, and linear correlations per cortical region) including source data for the main figures and the figures from the Supplementary Information data generated in this study have been deposited in a persistent repository of the University of Hamburg Center for Sustainable Research Data Management under accession code https://doi.org/10.25592/uhhfdm.16918 The raw MEG and MRI data are protected and are not publicly available due to data privacy laws. Raw MEG data may be shared upon request.

Research involving human participants, their data, or biological material

Policy information about studies with human participants or human data. See also policy information about sex, gender (identity/presentation), and sexual orientation and race, ethnicity and racism.

Reporting on sex and gender	16 males and 18 females were included in the study. Sex information was self-declared. Gender information was not collected. Sex-based analyses were not performed. Sex or gender differences were not considered in the study design. Sex-based difference was not the question of our study.
Reporting on race, ethnicity, or other socially relevant groupings	N/A
Population characteristics	34 healthy participants: age range 19-29 years. mean: 27 years, standard deviation: 5 years. 18 females.
Recruitment	34 participants were recruited from a database maintained by the Department of Neurophysiology and Pathophysiology at the University Medical Center Hamburg-Eppendorf. All participants received remuneration for their participation in the form of an hourly rate, and a study completion bonus.<br><RIsk of potential bias in sample selection><br>Recruitment was handled by an employee at the department of Neurophysiology and Pathophysiology at the University Medical Center Hamburg-Eppendorf. The employee was not involved in the study design nor was aware of the paradigm nor goal of the study. Therefore, we are confident there is no self-selection bias at the recruitment level. Moreover, the task itself involves simple perceptual decision -making, which is not expected to be affected by sub-samples of healthy young human adults.
Ethics oversight	The study was approved by the ethics committee of the Hamburg Medical Association. All participants provided written informed consent.

Note that full information on the approval of the study protocol must also be provided in the manuscript.

Field-specific reporting

Please select the one below that is the best fit for your research. If you are not sure, read the appropriate sections before making your selection.

☒ Life sciences ☐ Behavioural & social sciences ☐ Ecological, evolutionary & environmental sciences

For a reference copy of the document with all sections, see nature.com/documents/nr-reporting-summary-flat.pdf

Life sciences study design

All studies must disclose on these points even when the disclosure is negative.

Sample size	No power analysis was used prior to the study, because the effects of interest (dependence of stimulus encoding or readout in the brain) are unknown. We based our sample size of N=34 on those from previous MEG experiments using comparable stimulus and task designs (Murphy et al., Nature Neuroscience (2021) and Wilming et al., Nature Communications (2020)). Importantly, the chosen sample size was larger than the ones from any MEG experiment reported in these papers (ranging from N=15 to N=30), and we collected a large amount of data from each of the 34 participants in our experiment: a total of at least 1856 trials (distributed across 4 MEG sessions) from each participant.
Data exclusions	Four participants were excluded during the MEG preprocessing or source reconstruction stage (i.e., prior to the tests of the main experimental effects, see below), leaving N=30 participants for further data analyses. Three of those showed persistent MEG artifacts (of unclear origin) at the sensor-level, which could not be removed with the preprocessing pipeline described below; in a fourth, the source reconstruction (see below) failed for several parcels. One additional participant did not have a sufficient trial count for quantifying neural information measure separately for consistent and inconsistent conditions, leaving N=29 for consistency effects in neural data shown in Figures 4 and 5. We ensured that the behavioral consistency effects (i.e., on I(S;E)) shown in Figure 2 (panels a,b) were unaffected when

restricting the analysis to the same N=29 participants included in Figures 4 and 5.

Replication

This was not designed as a replication study. However, we applied a secondary analysis pipeline (linear regression) to the data, which reproduced the main findings of the primary analysis pipeline using information metrics.

Randomization

Randomization was not necessary since we had only one group of participants who all performed the same two behavioral tasks.

Blinding

Blinding was not necessary since we had only one group of participants who all performed the same two tasks.

Reporting for specific materials, systems and methods

We require information from authors about some types of materials, experimental systems and methods used in many studies. Here, indicate whether each material, system or method listed is relevant to your study. If you are not sure if a list item applies to your research, read the appropriate section before selecting a response.

Materials & experimental systems

n/a	Involved in the study
☒	☐ Antibodies
☒	☐ Eukaryotic cell lines
☒	☐ Palaeontology and archaeology
☒	☐ Animals and other organisms
☒	☐ Clinical data
☒	☐ Dual use research of concern
☒	☐ Plants

Methods

n/a	Involved in the study
☒	☐ ChIP-seq
☒	☐ Flow cytometry
☒	☐ MRI-based neuroimaging

Plants

Seed stocks

Report on the source of all seed stocks or other plant material used. If applicable, state the seed stock centre and catalogue number. If plant specimens were collected from the field, describe the collection location, date and sampling procedures.

Novel plant genotypes

Describe the methods by which all novel plant genotypes were produced. This includes those generated by transgenic approaches, gene editing, chemical/radiation-based mutagenesis and hybridization. For transgenic lines, describe the transformation method, the number of independent lines analyzed and the generation upon which experiments were performed. For gene-edited lines, describe the editor used, the endogenous sequence targeted for editing, the targeting guide RNA sequence (if applicable) and how the editor was applied.

Authentication

Describe any authentication procedures for each seed stock used or novel genotype generated. Describe any experiments used to assess the effect of a mutation and, where applicable, how potential secondary effects (e.g. second site T-DNA insertions, mosiacism, off-target gene editing) were examined.
